# Supplementary material for: Development and evaluation of a dual-target glycoconjugate against Shigella sonnei
Source: Front Immunol. 2026 Sep 21;17:1907725. doi: 10.3389/fimmu.2026.1907725 (PMC13635124; doi:10.3389/fimmu.2026.1907725)
Supplement: Supplementary file 1 [file Supplementaryfile1.docx]

Supplementary Figures and Tables

**Figure S1.** **Schematic of carrier protein design.** Each carrier protein (blue) was modified with a N-terminal DsbA signal sequence for trafficking to the periplasm (purple), double or triple glycine linkers (grey) and 6XHistag (red) for nickel-affinity purification. Carrier proteins varied by presence of ComP tag (green) for glycosylation with PglS. (A) C-terminal ComP tag and (B) no ComP tag.

**Figure S2.** **Western blot analysis of PglS-mediated glycosylation of ExoAComP with the *S. sonnei* O-antigen in *E. coli* SDB1.** (1) *S. sonnei* O-antigen (SSOAg) expression plasmid (pBPSO) with ExoAComP and PglS, (2), no PglS control- pBPSO and ExoAComP only, (3) no glycan control- ExoAComP and PglS only, (4) pBPSO co-expressed with PglB oligosaccharyltransferase and compatible carrier protein, ExoA10. Nickel-affinity purified proteins were resolved by SDS-PAGE and Western Blot probed using anti-6xHistag antibody (green) and anti-SSOAg antibody (red/yellow). White arrow indicates glycoconjugate.

**Figure S3.** **Densitometry analysis of *Shigella sonnei* glycoconjugate production.** Three biological replicates of ExoAComP, EmrKComP and MdtAComP glycosylated with the *S. sonnei* O-antigen (SSOAg) were resolved by SDS-PAGE and detected via Western blot using anti-6xHistag antibody (green) and anti- SSOAg antibody (red). Blue boxes denote regions used for glycoconjugate quantification via densitometry using ImageJ software.

**Figure S4.** **Sequential anion exchange chromatography (AEX) for purification of *S. sonnei-*specific glycoconjugates.** Nickel-affinity purified ExoAComP conjugated to the *S. sonnei* O-antigen (SSOAg) was further purified via AEX for separation of glycosylated and unglycosylated protein using an increasing NaCl gradient. Elution fractions from each round of AEX were resolved by SDS-PAGE and visualised by Western blot (top panel) and Imperial staining (bottom panel). Fractions containing the most glycoconjugate were pooled and AEX repeated. Images A to D represent sequential rounds of AEX, from first to last, with each well containing a different elution fraction. Western blots were visualized using anti-6xHistag antibody (green) and anti- SSOAg antibody (red).

**Figure S5. Anti-carrier protein antibody response to ExoA and MdtA, with and without ComP.** ELISA quantification (OD_450_ ) of IgG response to ExoA and MdtA, with and without ComP, in mice immunised with unglycosylated ExoAComP or MdtAComP, or PBS only. Each bar represents a different carrier protein, with pooled sera from each immunisation group on the x-axis. Error bars represent standard deviation from three technical replicates.

**Figure S6. Anti-carrier protein antibody response to ExoA and MdtA.** ELISA quantification of IgG response to ExoA and MdtA in mice immunised with glycosylated or unglycosylated (A) ExoAComP or (B) MdtAComP. Each line represents a different immunisation group. OD_450_ is reported for 6-point, 3-fold dilution series of sera, error bars represent standard deviation for seven biological replicates.

**Figure S7.** **Generation of W3110Δ*mdtA.*** *mdtA* was deleted from the W3110 chromosome using lambda red recombineering. *mdtA* was replaced with a chloramphenicol cassette which was flanked by *dif* recombinase sites for excision by the *E. coli* Xer recombinase. PCR using primers flanking the *mdtA* gene were used to screen colonies which had lost chloramphenicol resistance and amplicon resolved by gel electrophoresis. (1-4) W3110Δ*mdtA* clones, (5) W3110 wild-type, (6) dH_2_O negative control. Wild-type W3110, 1529 bp, W3110Δ*mdtA,* 309 bp.

**Figure S8.** Antibody response to *S. sonnei* O-antigen. ELISA quantification of IgG response to whole *E. coli* cells expressing *S. sonnei* O-antigen or empty vector (no O-antigen) in mice immunised with glycosylated or unglycosylated ExoAComP or MdtAComP. A 6-point, 3-fold dilution series was tested for pooled serum from each group and IgG response reported as the reciprocal serum dilution that gave an OD 0.2. Each represents a different immunisation group. Error bars represent standard deviation for three technical replicates. SSOAg, *S. sonnei* O antigen.

Supplemental Tables

**Table S1. List of strains used in this study.**

| **Strain** | **Genotype** | **Source** |
| --- | --- | --- |
| *E. coli* W3110 | F- *mcrA mcrB* In(*rrnD-rrnE*)1 | ATCC 27325 |
| *E. coli* W3110Δ*mdtA* |  | This study |
| *E. coli* SDB1 | W3110 Δ*waaL*, Δ*wecA* | (1) |
| *Plesiomonas shigelloides* O17 | O17 O-antigen | NCTC10360 |
| *E. coli* EC100D (*pir*+) | *F ^-^ mcrA Δ(mrr-hsdRMS-mcrBC) φ80dlacZΔM15 ΔlacX74 recA1 endA1 araD139 Δ(ara, leu)7697 galU galK λ- rpsL (Str ^R^) nupG pir ^+^(DHFR)* | (2) |
| *Shigella sonnei* 53G | *S. sonnei* laboratory reference strain | WRAIR |

**Table S2. List of plasmids used in this study.**

| **Plasmid** | **Description** | **Source** |
| --- | --- | --- |
| pBBR1MCS-3 | Cloning vector (Tet^R^) | (3) |
| pBPSO | pBBR1MCS-3 containing 12.2 Kb *Plesiomonas shigelloides* O17 O-antigen region from *wzz* to *wbgZ*, inserted into ApaI site. | This study |
| pACT3_pglS | pACT3 (Cm^R^) containing *Acinetobacter baylyi pglS* | (4) |
| pEXT22 | Expression vector (Kan^R^), IPTG-inducible | (7) |
| pEXT22_pglS | pEXT22 (Kan^R^) containing *Acinetobacter baylyi pglS,* inserted into EcoRI and BamHI sites. | This study |
| pEC415 | Expression vector (Amp^R^), L-arabinose inducible | (5) |
| pEC415_ExoAComP | pEC415 containing detoxified *Pseudomonas aeruginosa* ExoA with N-terminal DsbA periplasmic signal sequence and C-terminal ComP sequon and 6xHistag. | This study |
| pEC415_EmrKComP | pEC415 containing *Shigella* EmrK (amino acids 38-387) with N-terminal DsbA periplasmic signal sequence and C-terminal ComP sequon and 6xHistag. | This study |
| pEC415_MdtAComP | pEC415 containing *Shigella* MdtA (amino acids 66-453) with N-terminal DsbA periplasmic signal sequence and C-terminal ComP sequon and 6xHistag. | This study |
| pCH4 | pEXT20 containing detoxified *P. aeruginosa* ExoA with N-terminal DsbA periplasmic signal sequence and C-terminal ComP sequon and 6xHistag. | (6) |
| pEXT20 | Expression vector (Amp^R^), IPTG-inducible | (7) |
| pEXT20_ExoA | pEXT20 containing detoxified *P. aeruginosa* ExoA with N-terminal DsbA periplasmic signal sequence and 6xHistag. | This study |
| pEXT20_MdtA | pEXT20 containing *Shigella* MdtA (amino acids 66-453) with N-terminal DsbA periplasmic signal sequence and 6xHistag. | This study |
| pKD46 | Cloning vector (Amp^R^), containing lambda red recombineering machinery, temperature sensitive. | (8) |
| pKD4 | Cloning vector (Amp^R^, Kan^R^) used for construction of *mdtA* mutagenesis cassette. | (8) |
| pKD4_mdtA | pKD4 containing *mdtA* mutagenesis cassette. | This study |

**Table S3. List of oligonucleotides used in this study.**

| **Oligonucleotide** | **Description** | **Sequence** |
| --- | --- | --- |
| PsOFApaI | Amplification of 12.2 Kb region of *P. shigelloides* O-antigen locus, from *wzz* to *wbgZ* | tatcatGGGCCCgcagttggcgatatcctgtt |
| PsORApaI |  | aatcatGGGCCCtcacaggcgtatgacattgc |
| pEC415DsbA_f | Amplification of pEC415 for insertion of *exoAComP*, *emrKComP*, and *mdtAComP* expression constructs | attgagaattcttgaagacgaaa |
| pEC415DsbA_r |  | ttcattatgttattcctccttattta |
| ExoA_NComPdel_f | Removal of N-terminal ComP sequon from pEC415_ExoAComP | gcggccgaggaggcattcgatttatggaatgaatgtgc |
| ExoA_NComPdel_r |  | tccgccctgcgccgcgct |
| EmrK_NComPdel_f | Removal of N-terminal ComP sequon from pEC415_EmrKComP | ggcggtgaactggaagacatgattagtacggat |
| EmrK_NComPdel_r |  | ctgcgccgcgctagcgctaaa |
| MdtA_NComPdel_f | Removal of N-terminal ComP sequon from pEC415_MdtAComP | ggcggtggccgtaacgactca |
| MdtA_NComPdel_r |  | ctgcgccgcgctagcgctaaa |
| pEXT20_noComP_f | Amplification of pEXT20 for insertion of *exoA* and *mdtA* without ComP | caagcttctgttttggcgg |
| pEXT20_noComP_r |  | gaattctgtttcctgtgtgaaat |
| pKD4MdtA_f | Amplification of pKD4 for insertion of *mdtA* mutagenesis cassette | atatggaccatggctaattcccatg |
| pKD4MdtA_r |  | gcatgcaagcttggcactg |
| MdtAcassette_f | Amplification of *mdtA* mutagenesis cassette from pKD4_mdtA | atgtgcccgtcattcagacg |
| MdtAcassette_r |  | ggcgcgataaccgataatccc |
| MdtAscreen_f | Screening to confirm *mdtA* deletion from *E. coli* W3110 chromosome | ctcgcaaatgtgcccgtcatt |
| MdtAscreen_r |  | ggatagtccacttccggcag |

Capital letters indicate restriction sites.

**Table S4. Terrific Broth Recipe**

| **Component** | **Amount in 1 l** |
| --- | --- |
| Tryptone | 12 g |
| Yeast Extract | 24 g |
| KH_2_PO_4_ | 1.16g |
| K_2_HPO_4_ | 6.27 g |
| Glycerol | 4 ml |

References

1.	Garcia-Quintanilla F, Iwashkiw JA, Price NL, Stratilo C, Feldman MF. Production of a recombinant vaccine candidate against *Burkholderia pseudomallei* exploiting the bacterial N-glycosylation machinery. Front Microbiol. 2014;5:381.

2.	Metcalf WW, Jiang W, Wanner BL. Use of the rep technique for allele replacement to construct new *Escherichia coli* hosts for maintenance of R6K gamma origin plasmids at different copy numbers. Gene. 1994;138(1-2):1–7.

3.	Kovach ME, Elzer PH, Hill DS, Robertson GT, Farris MA, Roop RM, 2nd, et al. Four new derivatives of the broad-host-range cloning vector pBBR1MCS, carrying different antibiotic-resistance cassettes. Gene. 1995;166(1):175–6.

4.	Duke JA, Paschall AV, Robinson LS, Knoot CJ, Vinogradov E, Scott NE, et al. Development and Immunogenicity of a Prototype Multivalent Group B *Streptococcus* Bioconjugate Vaccine. ACS Infect Dis. 2021;7(11):3111–23.

5.	Enggist E, Schneider MJ, Schulz H, Thöny-Meyer L. Biochemical and mutational characterization of the heme chaperone CcmE reveals a heme binding site. J Bacteriol. 2003;185(1):175–83.

6.	Harding CM, Nasr MA, Scott NE, Goyette-Desjardins G, Nothaft H, Mayer AE, et al. A platform for glycoengineering a polyvalent pneumococcal bioconjugate vaccine using *E. coli* as a host. Nat Commun. 2019;10(1):891.

7.	Dykxhoorn DM, St Pierre R, Linn T. A set of compatible tac promoter expression vectors. Gene. 1996;177(1-2):133–6.

8.	Datsenko KA, Wanner BL. One-step inactivation of chromosomal genes in *Escherichia coli* K-12 using PCR products. Proc Natl Acad Sci U S A. 2000;97(12):6640–5.
